# Measuring mosquito control: adult-mosquito catches *vs* egg-trap data as endpoints of a cluster-randomized controlled trial of mosquito-disseminated pyriproxyfen

**DOI:** 10.1186/s13071-020-04221-z

**Published:** 2020-07-14

**Authors:** Klauss K. S. Garcia, Hanid S. Versiani, Taís O. Araújo, João P. A. Conceição, Marcos T. Obara, Walter M. Ramalho, Thaís T. C. Minuzzi-Souza, Gustavo D. Gomes, Elisa N. Vianna, Renata V. Timbó, Vinicios G. C. Barbosa, Maridalva S. P. Rezende, Luciana P. F. Martins, Glauco O. Macedo, Bruno L. Carvalho, Israel M. Moreira, Lorrainy A. Bartasson, Nadjar Nitz, Sérgio L. B. Luz, Rodrigo Gurgel-Gonçalves, Fernando Abad-Franch

**Affiliations:** 1 Núcleo de Medicina Tropical, Faculdade de Medicina, Universidade de Brasília, Brasilia, Brazil; 2 Secretaria de Vigilância em Saúde, Ministério da Saúde, Brasilia, Brazil; 3 Laboratório de Parasitologia Médica e Biologia de Vetores, Faculdade de Medicina, Universidade de Brasília, Brasilia, Brazil; 4 Laboratório Interdisciplinar de Biociências, Faculdade de Medicina, Universidade de Brasília, Brasilia, Brazil; 5 Diretoria de Vigilância Ambiental em Saúde, Subsecretaria de Vigilância à Saúde, Secretaria de Estado de Saúde do Distrito Federal, Brasilia, Brazil; 6 Instituto Leônidas e Maria Deane–Fiocruz Amazônia, Manaus, Brazil

**Keywords:** Mosquito-borne diseases, Mosquito control, Vector surveillance, Cluster randomized controlled trial, Pyriproxyfen

## Abstract

**Background:**

*Aedes aegypti* and *Culex quinquefasciatus* are the main urban vectors of arthropod-borne viruses causing human disease, including dengue, Zika, or West Nile. Although key to disease prevention, urban-mosquito control has met only limited success. Alternative vector-control tactics are therefore being developed and tested, often using entomological endpoints to measure impact. Here, we test one promising alternative and assess how three such endpoints perform at measuring its effects.

**Methods:**

We conducted a 16-month, two-arm, cluster-randomized controlled trial (CRCT) of mosquito-disseminated pyriproxyfen (MD-PPF) in central-western Brazil. We used three entomological endpoints: adult-mosquito density as directly measured by active aspiration of adult mosquitoes, and egg-trap-based indices of female *Aedes* presence (proportion of positive egg-traps) and possibly abundance (number of eggs per egg-trap). Using generalized linear mixed models, we estimated MD-PPF effects on these endpoints while accounting for the non-independence of repeated observations and for intervention-unrelated sources of spatial-temporal variation.

**Results:**

On average, MD-PPF reduced adult-mosquito density by 66.3% (95% confidence interval, 95% CI: 47.3–78.4%); *Cx. quinquefasciatus* density fell by 55.5% (95% CI: 21.1–74.8%), and *Ae. aegypti* density by 60.0% (95% CI: 28.7–77.5%). In contrast, MD-PPF had no measurable effect on either *Aedes* egg counts or egg-trap positivity, both of which decreased somewhat in the intervention cluster but also in the control cluster. Egg-trap data, therefore, failed to reflect the 60.0% mean reduction of adult *Aedes* density associated with MD-PPF deployment.

**Conclusions:**

Our results suggest that the widely used egg-trap-based monitoring may poorly measure the impact of *Aedes* control; even if more costly, direct monitoring of the adult mosquito population is likely to provide a much more realistic and informative picture of intervention effects. In our CRCT, MD-PPF reduced adult-mosquito density by 66.3% in a medium-sized, spatially non-isolated, tropical urban neighborhood. Broader-scale trials will be necessary to measure MD-PPF impact on arboviral-disease transmission. 
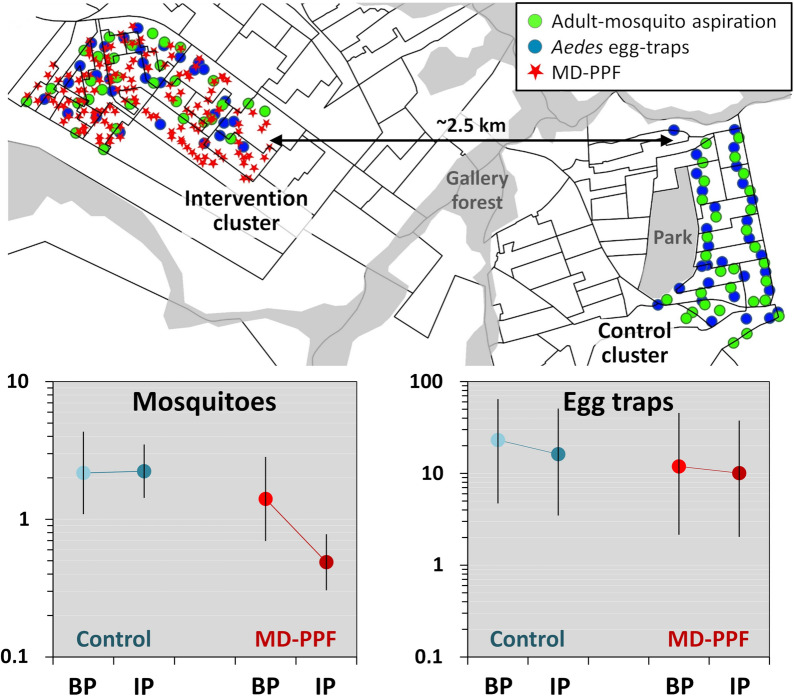

## Background

Urban mosquitoes are the main vectors of arthropod-borne viruses (arboviruses) causing disease in humans. In particular, *Aedes aegypti* and *Ae. albopictus* transmit, among other pathogens, the viruses causing dengue, Zika, chikungunya, or urban yellow fever, and members of the *Culex pipiens* complex including *Cx. quinquefasciatus* transmit (also among other pathogens) the viruses causing west Nile and Rift Valley fevers or Saint Louis encephalitis [1–4]. In the absence of specific antiviral treatments and (except for yellow fever and Japanese encephalitis) vaccines with true potential for broad public-health impact, mosquito control remains the cornerstone of arbovirus transmission control and arboviral-disease prevention [1, 5].

Although key to disease prevention, urban-mosquito control has met only limited success [1, 5, 6]. Traditional programmatic control tactics rely primarily on (i) treatment or removal/destruction of aquatic larval habitats by control agents and/or the public, which is hindered by the fact that detecting and gaining access to such habitats, especially the small, cryptic larval habitats of *Aedes* spp., can be very difficult; and (ii) adult-mosquito-targeted insecticide spraying, which often has only short-lived effects [5–14]. Among the several alternative control tactics under development/testing [6, 10], mosquito-disseminated pyriproxyfen (MD-PPF) specifically targets the challenge of larval-habitat detection and treatment [15–34]. It does so by attracting mosquitoes to surfaces coated with tiny PPF particles, which stick to the vectors’ bodies and are thus transferred by the mosquitoes themselves to otherwise untreated larval habitats [15–18].

PPF is an insect juvenile-hormone analogue that kills immature mosquitoes at minute doses and can be safely used in drinking water [35]. MD-PPF has yielded promising results in several semi-field and field trials based on the deployment of PPF ‘dissemination stations’, i.e. small artificial larval habitats that lure egg-laying mosquitoes and drive them to land/walk on PPF-coated surfaces. Most of the field trials reported to date, however, were too small-sized to provide useful operational guidance [18, 19, 21, 26, 27, 31], and the few trials conducted at the operationally-relevant scales of neighborhoods [25, 32] or towns [28] lacked truly independent controls. Two recent, moderately-sized (city block-scale), non-randomized trials were nominally controlled, but treatment and control blocks were spatially close to one another and, despite efforts to block migration, adult-mosquito exchange between blocks, including intervention ‘leakage’ into the control block, was likely [30, 33]. In sum, the lack of randomized, adequately controlled trials conducted at operationally relevant scales means that the evidence supporting dissemination-station-based MD-PPF as a useful means for urban-mosquito control remains relatively weak [6, 34, 36].

Here, we address this gap by presenting the results of a neighborhood-scale, parallel, two-arm cluster-randomized controlled trial (CRCT) of MD-PPF. In particular, we set to measure the impact of MD-PPF on local *Aedes* and *Culex* populations through both (i) active aspiration of adult mosquitoes, which directly measures adult-mosquito density [37–40], and (ii) egg-trap-based monitoring of female *Aedes* presence (proportion of positive egg-traps) and possibly abundance (number of eggs per egg-trap) [37, 41, 42]. Using a CRCT design, 16 months of field data, and a rigorous statistical-modeling strategy, we show that MD-PPF can significantly reduce adult-mosquito densities, yet *Aedes* egg-trap-based metrics may fail to detect this reduction.

## Methods

### Trial setting, design, and timeline

**Fig. 1:**
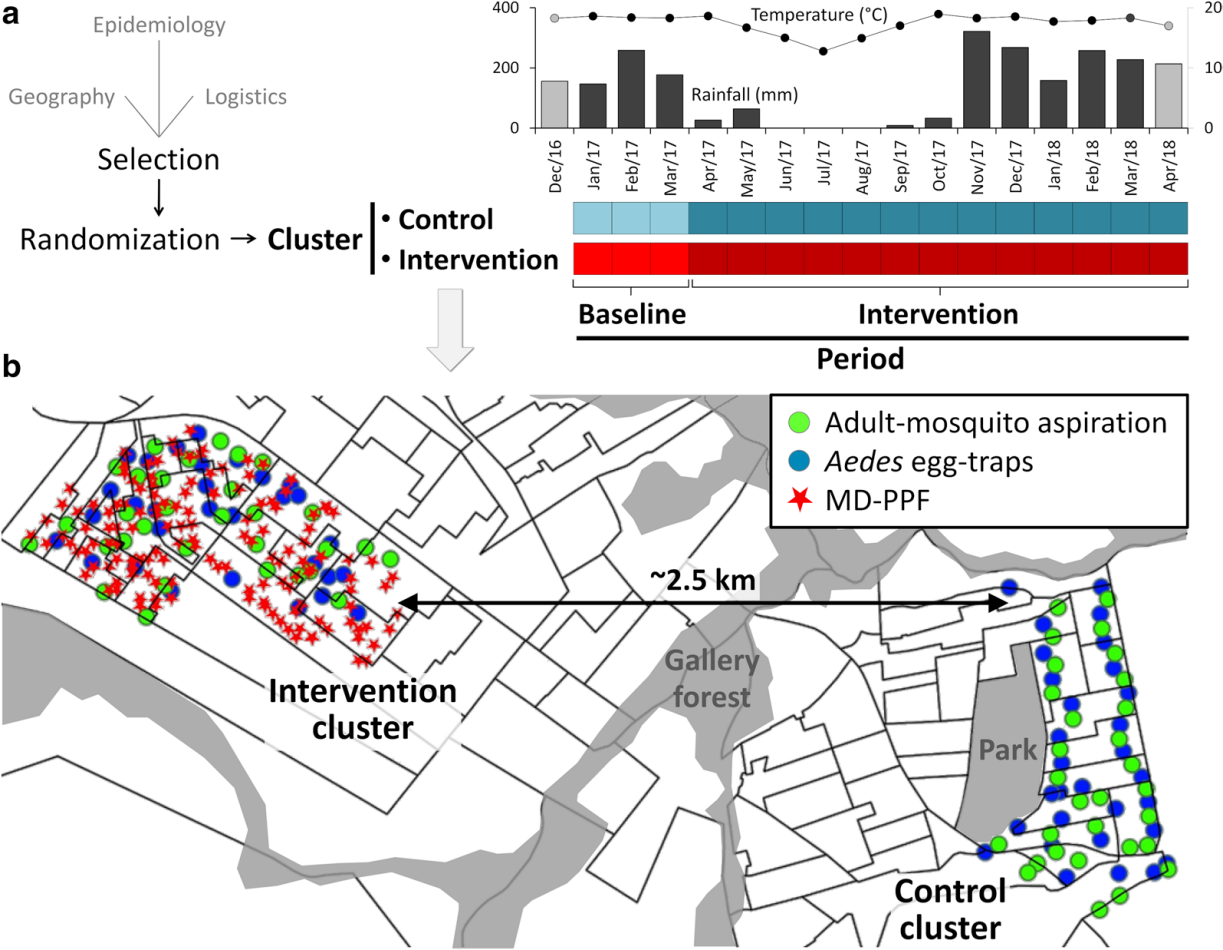
Trial setting, design, and timeline. Cluster selection and randomization plus trial timeline and overall weather conditions (rainfall and mean of daily minimum temperatures; weather conditions in December 2016 and April 2018 (lighter grey) were included only in some analyses) (**a**); and study clusters (**b**). One-hundred and fifty pyriproxyfen dissemination stations (red stars) were deployed over 13 months in the intervention cluster (**b**). Mosquito monitoring was run in both clusters from January 2017 to April 2018 (**a**) in 120 sampling dwellings: 60 for adult-mosquito catches with battery-powered aspirators (green dots) and 60 for *Aedes* egg-traps (blue dots) (**b**). *Abbreviation*: MD-PPF, mosquito-disseminated pyrpiproxyfen

This study took place in São Sebastião (15°54′36″S, 47°46′1″W), a lower-middle class, urban administrative region of the Federal District, Brazil. We combined map and satellite-image visual appraisal, field visits, and interviews with local health officials to select two residential clusters of similar sizes and urbanization patterns (mostly single-family homes with basic sanitation) for our CRCT. These two clusters (Fig. 1) met three main requirements: (i) reciprocal *geographical isolation*: about 2.5 km apart and separated by stretches of non-built environment (a gallery-forest patch and a forested park) to prevent or minimize intervention ‘leaking’ (i.e. dispersal of PPF-carrying mosquitoes) into the control cluster; (ii) *epidemiological similarity*: broadly comparable recent histories of arboviral-disease incidence, as judged by local health-surveillance officials; and (iii) *logistic feasibility*: manageable sizes (~ 1500–2000 homes each) given the project’s logistic and financial constraints. Once the two candidate clusters were selected, we tossed a coin to randomly assign one of them to the intervention (‘Intervention cluster’ in Fig. 1; IC hereafter), and retained the other as our ‘Control cluster’ (Fig. 1; CC hereafter). Mosquito monitoring was run from January 2017 to April 2018 in both clusters; in the IC, we deployed MD-PPF in April 2017 (see below). The CRCT therefore included a 3-month baseline period (BP) and a 13-month intervention period (IP); Fig. 1 includes a schematic of the trial timeline. In each cluster, we selected 30 dwellings for adult-mosquito catches and 30 different dwellings for *Aedes* egg-trap monitoring (Fig. 1). We aimed at achieving fair spatial coverage, with a reasonable number of sampling dwellings given logistic/financial constraints, within each cluster; therefore, we did not do *a priori* power/sample-size calculations and did not use randomization to select sampling dwellings.

### Adult-mosquito catch: aspiration

We used light-weight mosquito aspirators powered by 12-V, 5-Ah motorcycle batteries (Horst Armadilhas, São Paulo, Brazil) to collect adult mosquitoes; sampling took place once per month in each dwelling, with visits occurring between ~ 8–9 am and ~ 3–4 pm. After obtaining written informed consent, one field assistant dislodged mosquitoes from typical mosquito-resting surfaces (walls, curtains, plants, furniture, clothes, etc.) and a trained collector captured them with the aspirator. In each dwelling, aspiration covered the area around the house (including verandas/porches, patios, backyards, garages, gardens, etc.) and each room inside the house itself. At each sampling occasion, we recorded aspiration time to get a measure of mosquito-catch effort and noted the identity of the collector to check for possible among-collector variation in mosquito catches. Mosquitoes were stored in labeled (dwelling/date) pots, identified using standard keys [43], and counted.

### *Aedes* egg-trap monitoring

Each month, also after informed consent, we used two *Aedes* egg-traps, one indoors and one outdoors, in each sampling dwelling. *Aedes* egg-traps were 1-L, black plastic cups (12 cm in diameter) each fitted with a 10 × 3-cm, rough-surface fiberboard paddle suitable for *Aedes* egg-laying and filled with ~ 800 ml of tap water and ~80 ml of hay infusion. We set *Aedes* egg-traps in mosquito-prone locations and out of the reach of children or pets; after five days of operation, we collected the traps and used a stereomicroscope to count *Aedes* eggs present on each (trap/date-labeled) paddle.

### Intervention: mosquito-disseminated pyriproxyfen (MD-PPF)

We selected 150 dwellings of the IC to deploy, after getting written informed consent, one PPF ‘dissemination station’ in each from March 2017 to April 2018 (Fig. 1). These dwellings were roughly evenly distributed across the ~ 85.5-Ha IC; similar to [28], this yielded a density of about one dissemination station for every 10 dwellings (Fig. 1). PPF dissemination stations were 1.5-l, 15-cm diameter dark plastic cups filled with 400 ml of tap water; the inner wall was lined with black, Oxford-type cloth dusted with 5 g/m^2^ of PPF 0.5% (SumiLarv 0.5G® granules, Sumitomo Chemical, Tokyo, Japan) ground to talcum-like powder to allow dissemination by adult mosquitoes. We placed PPF dissemination stations in sun- and rain-protected spots out of the reach of children or pets, and serviced them (refilling water and re-dusting cloth with PPF) once per month; we also asked residents to check weekly that the cups contained water and to refill them if necessary. The CC remained without PPF dissemination stations (Fig. 1). We note that routine mosquito control by local health-surveillance agents was in place in both study clusters all through the trial. Control activities consisted primarily of active searches for larval habitats, which were physically destroyed or treated with SumiLarv 0.5G® PPF granules when detected; in addition, adult mosquito-killing ultra-low volume (ULV) malathion (aqueous emulsion 44%) was applied with truck-mounted sprayers (at a rate of 150 ml a.i./ha) when surveillance data suggested local transmission of *Aedes*-borne viruses [44, 45]. We recorded, for each dwelling and sampling occasion, whether ULV had been applied in the same city block (i) in the seven days before sampling (to test for immediate ULV effects), (ii) eight to 30 days before sampling (to test for lagged ULV effects), or (iii) > 30 days before sampling or never during the trial. This three-level factor variable (‘ulv’) allowed us to detect and, if present, adjust for ULV effects when assessing the MD-PPF effects of focal interest.

We finally note that, due to operational and logistic constraints, all field-team members had to be involved in both entomological monitoring and MD-PPF deployment; the trial, therefore, was cluster-randomized and controlled, but not blind.

### Data analysis

In an initial descriptive/exploratory step, we summarized our data using tables and graphs; we present counts, means, standard deviations, quantiles, or, when appropriate, proportions with score 95% confidence intervals (CI). In a second, inferential step, we used generalized linear mixed models (GLMMs; [46]) to estimate intervention (MD-PPF) effects on each endpoint (adult-mosquito density, as directly measured by aspiration; or female *Aedes* presence and possibly abundance, as indirectly measured by, respectively, egg-trap positivity and eggs per egg-trap) while accounting for:

(i) possible differences, unrelated to the intervention, between the IC and the CC;
(ii) the non-independence of measures taken at the same sampling dwellings in different months and during the same month in different dwellings;
(iii) possible temporal variation due to (a) monthly weather conditions (rainfall or temperature) or (b) ULV insecticide spraying; and
(iv) other, unmeasured sources of spatial or temporal variation.

All GLMMs contained a ‘cluster × period’ interaction, where ‘cluster’ is either the control cluster (CC) or the intervention cluster (IC) and ‘period’ is either the baseline period (BP) or the intervention period (IP) (Fig. 1); the slope coefficient estimated for the ‘IC × IP’ term measures the (link-scale) change in the endpoint variable that can be attributed to MD-PPF. All our models included, in addition, two random-intercept terms: one on dwelling ID to adjust for repeated measures, and one on study month to adjust for temporal variation not explained by fixed effects (e.g. in models including weather or ULV spraying; see below). The structure of the focal model was

$$\begin{aligned} & {\text{Y }}\sim {\text{ cluster }} \times {\text{ period }} + {\text{ weather }} + {\text{ ULV }} + {\text{ random}}\left( {\text{dwelling}} \right) \\ & \quad + {\text{ random}}\left( {\text{month}} \right) \, + {\text{ residual error}}, \\ \end{aligned}$$

where the endpoint ‘Y’ is the result of either adult-mosquito aspiration (a count) or *Aedes* egg-trap monitoring—an egg count or a binary 1/0 indicator of trap positivity/negativity.

Adult-mosquito catch models used the negative binomial distribution (log link-function) and further adjusted for sampling effort via an offset variable specifying the time (in 10-min units and log-transformed) spent in each sampling occasion; we, therefore, effectively modeled mosquito-catch rates per 10 min aspiration. We used this strategy to model the counts of, first, all mosquito species considered together, and, then, of *Ae. aegypti* and *Cx. quinquefasciatus* separately. In a supplementary set of analyses, we modeled female- and male-mosquito catches separately; the data, however, became too sparse to separately model sex-by-species strata. Egg-trap monitoring, on the other hand, yields information on *Aedes* egg density (eggs per egg-trap) and trap positivity/negativity. We jointly analyzed these two variables using two-part, zero-inflated models [47] with a binomial (logit link) submodel for the probability that a trap is *negative* and a negative binomial (log link) submodel for the egg counts. Both submodels had random-intercept terms on dwelling ID and month as described above.

We adjusted for weather-related temporal variation using data provided by the Brazilian Instituto Nacional de Meteorologia (www.inmet.gov.br). In particular, we built covariates measuring temperature (mean of daily minimum, mean, and maximum) and total rainfall in the week before each sampling occasion (‘tmin_w’, ‘tmean_w’, ‘tmax_w’, ‘rain_w’), in the week before that (i.e. one-week-lagged; ‘tmin_2w’ etc.), and in the month before each sampling occasion (‘tmin_m’ etc.; see Fig. 1 and Additional file 1: Table S1). We fitted GLMMs with one of these weather covariates (standardized to mean 0 and SD 1) at a time, compared model performance using the Bayesian information criterion (BIC), and selected the smallest-BIC model as our top-performing ‘full’ model for each outcome [48]. We then investigated the importance of ULV-spraying effects by removing the ‘ulv’ covariate from each top-performing ‘full’ model (or submodel in zero-inflated GLMMs) and then checking whether this removal improved or worsened model performance, i.e. whether it reduced or increased the model’s BIC score [48]. Finally, we checked for among-collector variation in mosquito-aspiration results by refitting the top-performing *Aedes* + *Culex* model with an extra random term (‘collector’) and comparing both specifications using BIC [48].

We did all our analyses in R 3.6.3 [49], using packages *stats* 3.6.3 [49], *Hmisc* 4.3-1 [50], *glmmTMB* 1.0.1 [51], *AICcmodavg* 2.2-2 [52] and *bbmle* 1.0.23.1 [53]. We report, for each analysis, both the numerical results of the smallest-BIC model and the predictions of that model (at selected covariate values) computed with *ggeffects* 0.14.2 [54]; given our focus on population-level intervention effects, we present estimated marginal means with 95% *confidence* intervals—not *prediction* intervals, which take random-effect variances into account [54]. For completeness, below we will also comment on the results of selected, non-top-ranking GLMMs of special interest, in particular, models including ULV effects. All our analyses are on an intention-to-treat basis, i.e. disregarding the occasional malfunctioning of some (5.6% overall) dissemination stations.

## Results

### Adult-mosquito catch: aspiration

We gathered data in 957 sampling occasions (dwelling-month aspiration events) totaling 10,267 min of adult-mosquito aspiration; mean effort was 10.7 min (SD = 4.6; median = 10.0 min) of aspiration per sampling occasion. Overall, we caught 4356 adult mosquitoes (783 *Ae. aegypti* and 3573 *Cx. quinquefasciatus*; Table 1). Raw data are available in Additional file 2: Dataset S1.

**Table 1:** Adult-mosquito catches using battery-powered aspirators: summary statistics

Metric	Statistic	CC			IC			Total
		BP	IP	Subtotal	BP	IP	Subtotal	
Sampling effort (in 60 dwellings over 16 months)								
Sampling occasions	Sum	90	388	478	90	389	479	957
Minutes of aspiration	Sum	1049	4325	5374	859	4034	4893	10,267
All-mosquito catches (*Aedes* + *Culex*)								
Total caught	Sum	620	2856	3476	412	468	880	4356
Mosquitoes per 10 min aspiration^a^	Mean	**5.81**	**5.09** (− 12%)^b^	5.22	**3.63**	**1.14** (− 69%)^b^	1.60	3.41
	SD	12.96	18.84	17.87	8.65	3.92	5.23	13.28
	Median	2.11	1.25	1.43	1.11	0.00	0.00	0.00
	IQR	1.00–5.00	0.00–4.00	0.00–4.53	0.00–3.56	0.00–0.91	0.00–1.25	0.00–2.50
	Maximum^c^	98.75	322.50	322.50	74.17	43.33	74.17	322.50
*Aedes aegypti* catches								
Total caught	Sum	94	217	311	275	197	472	783
*Aedes* per 10 min aspiration^a^	Mean	**0.99**	**0.52** (− 47%)^b^	0.61	**2.54**	**0.48** (− 81%)^b^	0.87	0.74
	SD	1.74	1.29	1.40	8.13	1.75	3.93	2.95
	Median	0.25	0.00	0.00	0.00	0.00	0.00	0.00
	IQR	0.00–1.33	0.00–0.43	0.00–0.76	0.00–2.00	0.00–0.00	0.00–0.50	0.00–0.67
	Maximum^c^	11.67	10.00	11.67	72.50	23.33	72.50	72.50
*Culex quinquefasciatus* catches								
Total caught	Sum	526	2639	3165	137	271	408	3573
*Culex* per 10 min aspiration^a^	Mean	**4.82**	**4.57** (− 5%)^b^	4.62	**1.09**	**0.66** (− 39%)^b^	0.74	2.68
	SD	12.32	18.70	17.67	2.40	2.94	2.85	12.79
	Median	1.43	0.56	0.72	0.00	0.00	0.00	0.00
	IQR	0.00–4.47	0.00–3.08	0.00–3.33	0.00–1.11	0.00–0.00	0.00–0.00	0.00–1.67
	Maximum^c^	96.25	322.50	322.50	15.38	38.00	38.00	322.50

^a^Values computed across the results of individual sampling occasions

^b^The percent change in mean mosquito catch (highlighted in bold typeface) between the baseline period and the intervention period is given in parentheses; note that, although the change was always a decrease (hence the minus signs), the decrease was always much larger in the IC than in the CC

^c^In all cases, the minimum number of adult mosquitoes caught per 10 min aspiration was zero

*Abbreviations*: CC, control cluster; IC, intervention cluster; BP, baseline period; IP, intervention period; SD, standard deviation; IQR, inter-quartile range

#### All mosquitoes

**Fig. 2:**
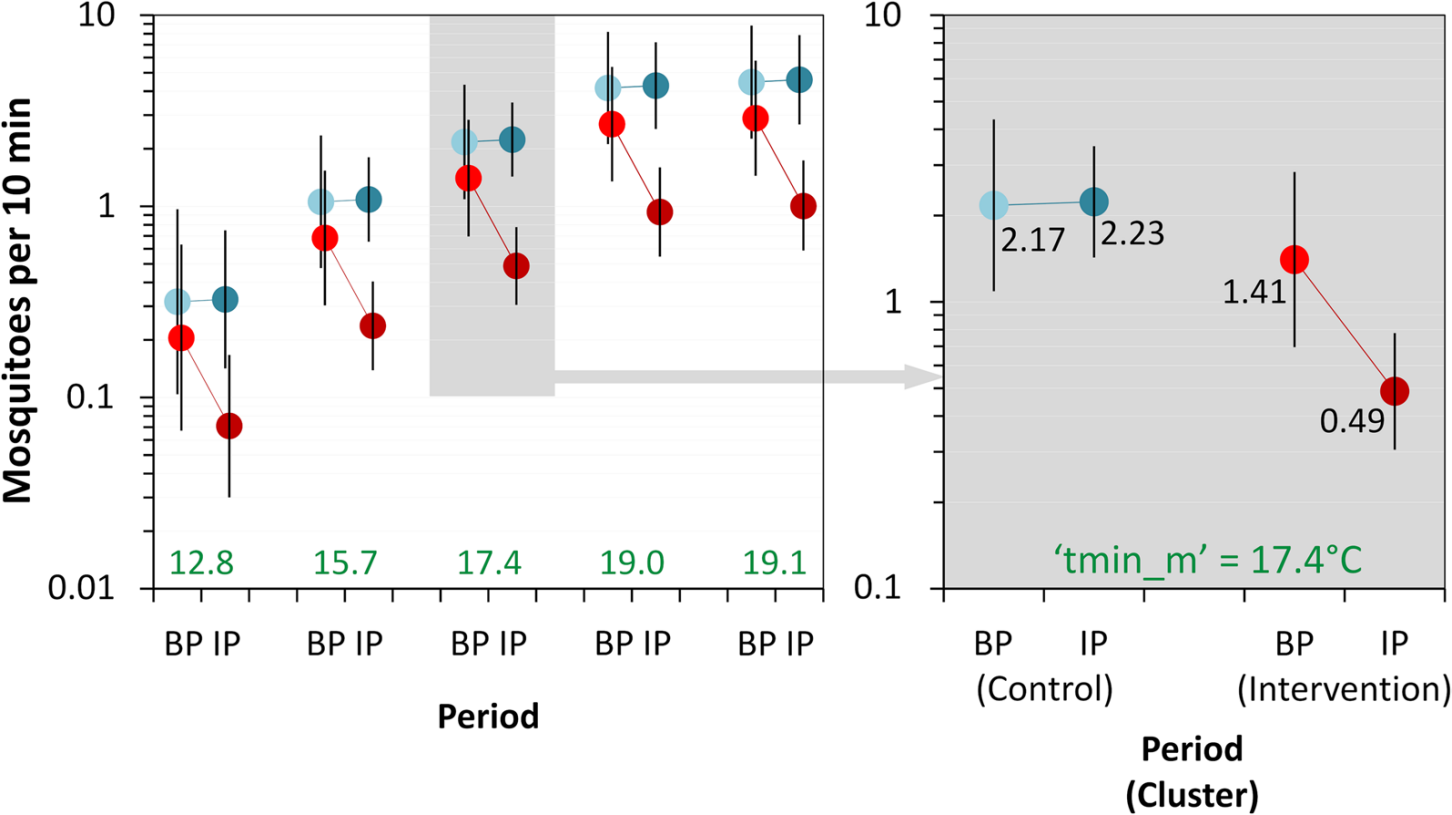
Effects of mosquito-disseminated pyriproxyfen on adult-mosquito catches (*Aedes aegypti* + *Culex quinquefasciatus*). Predictions of the top-ranking generalized linear mixed model at selected values (in green font) of covariate ‘tmin_m’ (mean of daily minimum temperatures in the month before sampling) across trial clusters (blue, control cluster; red, intervention cluster) and periods (lighter, baseline period; darker, intervention period). The right-hand panel shows in greater detail the predictions of the model at the observed mean ‘tmin_m’ value (17.4 °C). *Abbreviations*: BP, baseline period; IP, intervention period

The average catch over sampling occasions was 3.41 ± 13.28 SD adult mosquitoes per 10 min aspiration (Table 1). At baseline, mosquito density appeared to be higher in the CC (5.81) than in the IC (3.63). In the IC, mean density fell by 68.6% (to 1.14 mosquitoes per 10 min aspiration) during the intervention, whereas density barely changed in the CC (Table 1). Table 1 presents a summary of observations, overall and stratified by cluster and period; for monthly results see Additional file 1: Table S2. Our BIC-based assessment selected average minimum temperatures in the month before sampling (‘tmin_m’) as the best-fitting weather covariate (Additional file 1: Table S3). This smallest-BIC ‘full’ model, however, performed substantially worse (BIC difference 10.1 units) than a simpler, alternative model excluding ULV effects (Table 2 and Additional file 1: Table S3). After adjusting for dwelling-level repeated measures and random month-to-month variation, as well as for the (positive) effect of warmer nights (as measured by ‘tmin_m’) and for intervention-unrelated differences between clusters, this model estimates a clearly (*sensu* [55]) negative effect of MD-PPF on adult-mosquito catches: *β*_IC×IP_ = − 1.086, 95% CI: − 1.532 to − 0.641 (Table 2). This is equivalent to an incidence rate ratio *e*^−1.086^ = 0.337, which indicates that the intervention brought about a 100 − 33.7 = 66.3% reduction (95% CI: 47.3–78.4%) of mean adult-mosquito density (Table 2). Figure 2 shows the predictions of this top-performing model for selected ‘tmin_m’ values across trial clusters and periods. Adding the ‘collector’ random term to this model increased its BIC score by 5.8 units (Additional file 1: Table S3); among-collector variation was very small (SD = 0.13), and the *β*_IC×IP_ estimate (− 1.030 ± 0.247 SE) was similar to that of the top-ranking model. We finally note that the ‘full’ model including ‘ulv’ estimated a nearly-zero lagged ULV effect (*β*_ULVlag_ = 0.020 ± 0.166 SE) and a negative, yet imprecise, immediate ULV effect (*β*_ULVweek_ = − 0.936; 95% CI: − 1.880–0.007); the effect of MD-PPF remained clearly [55] negative (*β*_IC×IP_ = − 1.075 ± 0.243 SE) after ULV adjustment (see Additional file 1: Table S4). These results broadly mirrored those of modeling male and female mosquito catches separately; effect estimates from the top-ranking models were *β*_IC×IP_ = − 0.715 ± 0.242 SE for females and *β*_IC×IP_ = − 1.451 ± 0.297 SE for males (see details in Additional file 3: Tables S11 and S12).

**Table 2:** Adjusted effects of mosquito-disseminated pyriproxyfen on adult-mosquito catches (*Aedes aegypti* + *Culex quinquefasciatus*): top-ranking (smallest-BIC) generalized linear mixed model

Term	Estimate	SE	95% CI	
			Lower	Upper
Fixed effects				
Intercept (CC, BP)^a^	0.776	0.352	0.087	1.465
Intervention period (IP)^b^	0.028	0.341	− 0.641	0.697
Intervention cluster (IC)	− 0.436	0.317	− 1.056	0.185
IP × IC^c^	− 1.086	0.227	− 1.532	− 0.641
Temperature^d^	0.721	0.139	0.448	0.994
Random effects SD				
Dwelling ID	0.956	–	0.773	1.183
Month	0.456	–	0.303	0.686

^a^The intercept estimates the (log-scale) expected mean number of mosquitoes caught per 10 min aspiration in the CC, in the typical dwelling and at typical temperatures, during the BP; the other fixed-effect slope coefficients estimate changes in this expectation associated with period, cluster, intervention, and temperature effects

^b^Note that the model estimates a near-zero change in (log) mean mosquito-catch as the CC entered the IP (but received no intervention); the estimated incidence rate ratio is *e*^0.028^ = 1.028, or a 2.8% increase in mean mosquito-catch, with the 95% CI spanning zero

^c^The ‘IP × IC’ interaction coefficient estimates the (log) change in expected mean mosquito-catch that can be attributed to the intervention – deployment of 150 pyriproxyfen dissemination stations over 13 months (the intervention period ‘IP’) in the intervention cluster ‘IC’. Here, the model estimates an *e*^− 1.086^ = 0.337 incidence rate ratio, indicating that the intervention resulted in a 100 −  33.7 = 66.3% reduction (95% CI: 47.3–78.4%) of the expected mean mosquito-catch

^d^Specified as the (standardized) mean of minimum daily temperatures in the month before each sampling occasion (‘tmin_m’); the original variable had mean = 17.39°C and SD = 1.73°C. Given our focus on estimating adjusted intervention effects, we considered weather covariates as confounders; ‘tmin_m’ yielded better-performing models, as measured by BIC scores, than other measures of temperature and rainfall

*Abbreviations*: BIC, Bayesian information criterion; SE, standard error; 95% CI, 95% confidence interval (lower/upper limits); CC, control cluster; BP, baseline period; IP, intervention period; IC, intervention cluster; SD, standard deviation; ID, identity of each sampling dwelling

#### *Aedes aegypti* adults

**Fig. 3:**
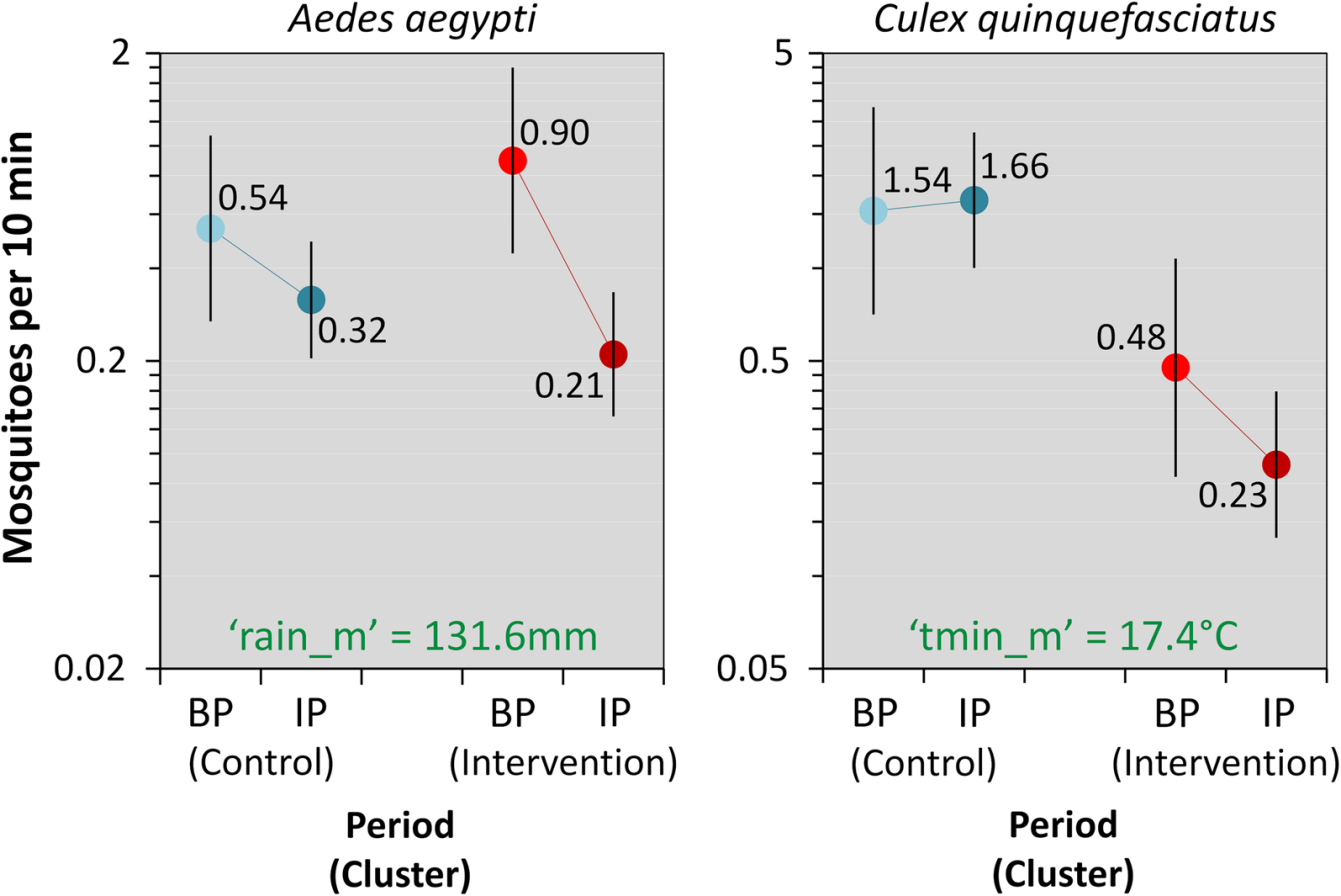
Effects of mosquito-disseminated pyriproxyfen on species-specific adult-mosquito catches (*Aedes aegypti* and *Culex quinquefasciatus*). Predictions of the top-ranking generalized linear mixed models at mean values (in green font) of covariates ‘rain_m’ (total rainfall in the month before sampling) and ‘tmin_m’ (mean of daily minimum temperatures in the month before sampling) across trial clusters (blue, control cluster; red, intervention cluster) and periods (lighter, baseline period; darker, intervention period). *Abbreviations*: BP, baseline period; IP, intervention period

At baseline, catches of adult *Ae. aegypti* were particularly productive in the IC; during the intervention period, the mean catch per 10 min aspiration fell by 81.1% in the IC and by 47.5% in the CC (Table 1; see also Additional file 1: Table S2). BIC scores identified rainfall in the month before sampling (‘rain_m’) as the best-fitting weather covariate; the performance of this rainfall ‘full’ model was again improved, albeit by a small amount (BIC difference 0.98 units), by removing ULV effects (Additional file 1: Table S5). The top-ranking *Ae. aegypti* model (Table 3) estimates a positive effect of rainfall and a negative effect of MD-PPF as measured by the ‘IC × IP’ slope coefficient, with a 60.0% decrease (95% CI: 28.7–77.5%) of mean *Aedes* catches attributable to the intervention (Table 3). Figure 3 presents a selected subset of this model’s predictions. The ‘full’ model including ULV effects suggests that, relative to no recent ULV spraying, mean *Aedes* catch was actually *higher* when ULV had been applied between 8 and 30 days before sampling (*β*_ULVlag_ = 0.607 ± 0.193 SE), but lower when ULV had been applied during the week before sampling (*β*_ULVweek_ = − 1.061 ± 0.511 SE); MD-PPF still had an overall negative, ULV-adjusted effect on adult *Ae. aegypti* density (a 44.7% decrease), but the 95% CI ran from a slight (1.92%) increase to a clear (70.0%) decrease (Additional file 1: Table S6).

**Table 3:** Adjusted effects of mosquito-disseminated pyriproxyfen on species-specific adult-mosquito catches (*Aedes aegypti* and *Culex quinquefasciatus*): top-ranking (smallest-BIC) generalized linear mixed models

Term	Estimate	SE	95% CI	
			Lower	Upper
*Aedes aegypti*				
Fixed effects				
Intercept (CC, BP)^a^	− 0.618	0.354	− 1.312	0.077
Intervention period (IP)^b^	− 0.535	0.367	− 1.253	0.184
Intervention cluster (IC)	0.508	0.319	− 0.118	1.134
IP × IC^c^	− 0.916	0.295	− 1.493	− 0.338
Rainfall^d^	0.829	0.146	0.543	1.116
Random effects SD				
Dwelling ID	0.766	–	0.585	1.001
Month	0.455	–	0.275	0.755
*Culex quinquefasciatus*				
Fixed effects				
Intercept (CC, BP)^a^	0.430	0.396	− 0.346	1.205
Intervention period (IP)^b^	0.080	0.382	− 0.669	0.828
Intervention cluster (IC)	− 1.172	0.380	− 1.917	− 0.427
IP × IC^c^	− 0.807	0.291	− 1.378	− 0.237
Temperature^d^	0.707	0.156	0.400	1.012
Random effects SD				
Dwelling ID	1.091	–	0.868	1.370
Month	0.502	–	0.328	0.767

^a^The intercept estimates the (log-scale) expected mean number of mosquitoes caught per 10 minutes aspiration in the CC, in the typical dwelling and at typical temperatures, during the BP; the other fixed-effect slope coefficients estimate changes in this expectation associated with period, cluster, intervention, and rainfall or temperature effects

^b^Note that both models estimate non-significant changes in (log) mean mosquito-catch as the CC entered the IP (but received no intervention), with the 95% confidence intervals including zero

^c^The ‘IP × IC’ interaction coefficients estimate the (log) change in expected mean mosquito-catch that can be attributed to the intervention (deployment of 150 pyriproxyfen dissemination stations over 13 months (the IP) in the IC). The *Aedes* model estimates an *e*^− 0.916^ = 0.400 incidence rate ratio, indicating that the intervention resulted in a 100 − 40.0 = 60.0% reduction (95% CI: 28.7–77.5%) of the expected mean *Aedes*-catch; the *Culex* model estimates an *e*^− 0.807^ = 0.446 incidence rate ratio, or a 55.4% reduction (95% CI: 21.1–74.8%) of the expected mean *Culex* catch

^d^Specified as the (standardized) total rainfall in the month before sampling (‘rain_m’) for the *Aedes* model and as the mean of minimum daily temperatures in the month before sampling (‘tmin_m’) for the *Culex* model; the original variables had the following means (SDs): ‘rain_m’, 131.6 mm (111.3); ‘tmin_m’, 17.39°C (1.73). Given our focus on estimating adjusted intervention effects, we considered weather covariates as confounders; those in the table yielded better-performing models, as measured by BIC scores, than other measures of temperature and rainfall

*Abbreviations*: BIC, Bayesian information criterion; SE, standard error; 95% CI, 95% confidence interval (lower/upper limits); CC, control cluster; BP, baseline period; IP, intervention period; IC, intervention cluster; SD, standard deviation; ID, identity of each sampling dwelling

#### *Culex quinquefasciatus* adults

The density of adult southern house mosquitoes was particularly high in the CC both at baseline and during the intervention period; in the IC, mean adult *Culex* catches were 39.5% lower during MD-PPF deployment than at baseline (Table 1 and Additional file 1: Table S2). Our BIC-based assessment selected ‘tmin_m’ as the best-fitting weather covariate. However, the *Culex* GLMM that did not include ULV-spraying effects clearly outperformed (BIC difference 11.9 units) the ‘full’ model with both ‘tmin_m’ and ‘ulv’ (Additional file 1: Table S7). This top-performing model (Table 3) suggests, after adjusting for multiple additional sources of variation, that MD-PPF reduced adult *Cx. quinquefasciatus* density by about 55.4% (95% CI: 21.1–74.8%) (see Table 3 and Fig. 3). The ‘full’ *Culex* model estimates a near-zero lagged ULV effect (*β*_ULVlag_ = 0.009 ± 0.204 SE), a negative but imprecise immediate ULV effect (*β*_ULVweek_ = − 0.769 ± 0.561 SE), and a negative effect of MD-PPF deployment (*β*_IC×IP_ = − 0.801 ± 0.314 SE) (Additional file 1: Table S8).

### *Aedes* egg-trap monitoring

**Fig. 4:**
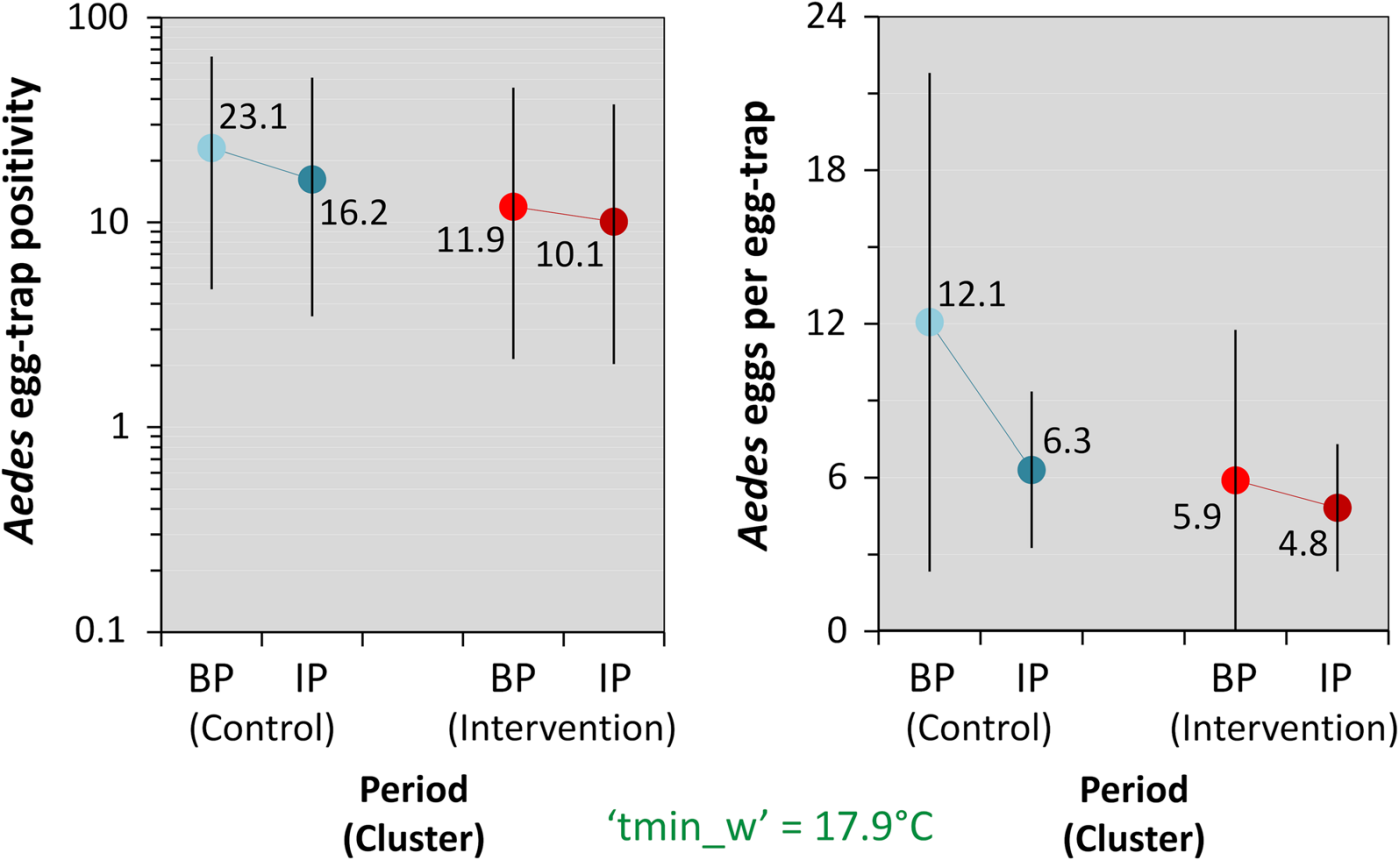
Effects of mosquito-disseminated pyriproxyfen on *Aedes* egg-trap-derived endpoints. Predictions of the top-ranking zero-inflated generalized linear mixed model at mean values (in green font) of covariate ‘tmin_w’ (mean of daily minimum temperatures in the week before sampling) across trial clusters (blue, control cluster; red, intervention cluster) and periods (lighter, baseline period; darker, intervention period). *Abbreviations*: BP, baseline period; IP, intervention period

We gathered data from a total of 1879 *Aedes* egg-traps-week; the main descriptive results are summarized in Table 4. At baseline, both *Aedes* egg-trap positivity and mean *Aedes* egg counts per egg-trap were somewhat lower in the IC than in the CC (Table 4), despite substantially higher mean adult *Aedes* catches in the latter (Table 1). When comparing the baseline and intervention periods, the data suggest that *Aedes* egg-trap-based metrics decreased somewhat in the CC but remained largely stable (perhaps with a slight decline of egg-trap positivity) in the IC (Table 4; see Additional file 1: Table S9 for monthly results and Additional file 2: Dataset S1 for the raw data). Our BIC-based assessment selected ‘tmin_w’ as the best-fitting weather covariate; the top-performing ‘full’ model including also ULV effects (in both the egg-count and the trap-negativity submodels) was again outperformed by simpler models lacking ‘ulv’ in either submodel, and these models, in turn, were clearly outperformed (BIC differences > 9 units) by an even simpler model excluding ULV effects from both the negative binomial (egg-count) and the binomial (trap-negativity) submodels (Additional file 1: Table S10). This top-ranking model suggests (after multiple adjustments as noted above) that there were no measurable cluster, period, or MD-PPF effects on *Aedes* egg counts per egg-trap, which were however clearly higher when nights were warmer; the binomial submodel, on the other hand, predicts lower egg-trap positivity in the IC (11.9% at baseline, 10.1% during the intervention) than in the CC (23.1% and 16.2%, respectively) (Table 5 and Fig. 4).

**Table 4:** Egg-trap based monitoring of *Aedes aegypti*: summary statistics

Metric	Statistic	CC			IC			Total
		BP	IP	Subtotal	BP	IP	Subtotal	
Sampling effort (in 60 dwellings over 16 months)								
Egg-traps set	Sum	168	767	935	167	777	944	1879
*Aedes* egg-trap positivity								
Egg-traps positive	Sum	54	172	226	32	130	162	388
	Percent	**32.14**	**22.43** (− 30%)^a^	24.17	**19.16**	**16.73** (− 13%)^a^	17.16	20.65
	95% CI (lower)	25.55	19.62	21.54	13.91	14.27	14.89	18.88
	95% CI (upper)	39.54	25.51	27.02	25.8	19.52	19.70	22.54
*Aedes aegypti* eggs in egg-traps								
Total eggs	Sum	3619	13,617	17,236	2224	10,931	13,155	30,391
Eggs per egg-trap^b^	Mean	**21.54**	**17.75** (− 18%)^a^	18.43	**13.32**	**14.07** (+ 6%)^a^	13.93	16.17
	SD	57.27	55.34	55.68	51.46	44.60	45.86	51.02
	Median	0	0	0	0	0	0	0
	IQR	0–20.25	0–0	0–0	0–0	0–0	0–0	0–0
	Maximum^c^	567	506	567	567	465	567	567

^a^The percent change in each key metric (highlighted in bold typeface) between the baseline period and the intervention period is given in brackets; trap positivity decreased moderately in both in the IC and in the CC, and eggs per egg-trap decreased in the IC but increased slightly in the CC

^b^Values computed across the results of individual sampling occasions

^c^In all cases, the minimum number of *Aedes aegypti* eggs per egg-trap was zero

*Abbreviations*: CC, control cluster; IC, intervention cluster; BP, baseline period; IP, intervention period; 95% CI, 95% score confidence interval (lower/upper limits); SD, standard deviation; IQR, inter-quartile range

**Table 5:** Adjusted effects of mosquito-disseminated pyriproxyfen on *Aedes* egg-trap-derived endpoints: numerical results of the top-ranking (smallest-BIC) zero-inflated generalized linear mixed model

Term	Estimate	SE	95% CI	
			Lower	Upper
Egg-count submodel				
Fixed effects				
Intercept (CC, BP)^a^	3.957	0.203	3.558	4.355
Intervention period (IP)	− 0.296	0.225	− 0.736	0.145
Intervention cluster (IC)	− 0.055	0.229	− 0.504	0.394
IP × IC	0.262	0.244	− 0.215	0.740
Temperature^b^	0.695	0.113	0.473	0.917
Random effects SD				
Dwelling ID	0.270	-	0.152	0.478
Month	0.244	-	0.115	0.519
Egg-trap *negativity* submodel				
Fixed effects				
Intercept (CC, BP)^c^	1.204	0.404	0.412	1.996
Intervention period (IP)	0.440	0.436	− 0.414	1.293
Intervention cluster (IC)	0.797	0.312	0.186	1.407
IP × IC	− 0.251	0.311	− 0.861	0.360
Temperature^b^	− 1.255	0.200	− 1.646	− 0.863
Random effects SD				
Dwelling ID	0.576	–	0.403	0.823
Month	0.593	–	0.384	0.916

^a^The intercept of the negative binomial (egg count) submodel estimates the (log-scale) expected mean number of *Aedes* eggs per egg-trap in CC, in the typical dwelling and at typical temperatures, during the BP; the other fixed-effect slope coefficients estimate changes in this expectation associated with period, cluster, intervention, and temperature effects; only this latter was clearly (*sensu* [55]) different from zero

^b^Specified as the (standardized) mean of minimum daily temperatures in the week before each sampling occasion (‘tmin_w’); the original variable had mean = 17.86 °C and SD = 2.89 °C. Given our focus on estimating adjusted intervention effects, we considered weather covariates as confounders; ‘tmin_w’ yielded better-performing models, as measured by BIC scores, than other measures of temperature and rainfall

^c^The intercept of the binomial (egg-trap negativity) submodel estimates the (logit-scale) expected proportion of *negative* egg-traps in the CC, in the typical dwelling and at typical temperatures, during the BP; the other fixed-effect slope coefficients estimate changes in this expectation associated with period, cluster, intervention, and temperature effects – with results suggesting higher baseline odds of egg-trap *negativity* in the CC and that warmer nights were independently associated with lower odds of egg-trap *negativity*

*Abbreviations*: BIC, Bayesian information criterion; SE, standard error; 95% CI, 95% confidence interval (lower/upper limits); CC, control cluster; BP, baseline period; IP, intervention period; IC, intervention cluster; SD, standard deviation; ID, identity of each sampling dwelling

## Discussion

In this report, we present results of a parallel, two-arm, cluster-randomized controlled trial (CRCT) of mosquito-disseminated pyriproxyfen (MD-PPF); our study yielded two key findings and an additional, potentially useful insight. First, our MD-PPF-based intervention clearly reduced (by 66.3% on average) adult-mosquito density in a medium-sized, spatially non-isolated, lower-middle class urban neighborhood of central Brazil. Secondly, trial endpoints based on *Aedes* egg-trap monitoring failed to capture this reduction, which reached 60.0% on average for adult *Ae. aegypti*. Finally, our analyses indicate that pulses of ultra-low volume malathion spraying (ULV) had at most a very transient (about one-week) effect on local adult-mosquito density, with, again, no measurable impact on *Aedes* egg-trap-derived metrics.

Our CRCT provided strong evidence [36] that MD-PPF can considerably reduce adult-mosquito density at the neighborhood scale. Using rigorous statistical modeling to adjust for multiple potential sources of spatial-temporal variation (see Tables 2, 3 and 5, Additional file 1: Tables S1–S10, and “Methods”), we show that mean adult-mosquito density was nearly three times higher before than during MD-PPF deployment in the residential cluster randomized to receive the intervention; in contrast, adult-mosquito density remained stable across periods in the control cluster (Fig. 2). This key result was consistent across datasets (Figs. 2 and 3, Tables 2 and 3), suggesting broadly comparable intervention effects on adult *Ae. aegypti* and *Cx. quinquefasciatus*, the two most important urban vectors of disease-causing arboviruses [1–4].

Putting these results in the context of previous findings is at best problematic. Even in the case of field trials using PPF dissemination stations and measuring adult-mosquito density at scales similar to ours (city block or neighborhood), methodological issues complicate direct comparisons. For example, the clear reduction of adult-mosquito density we report is larger than found in similarly-sized MD-PPF field trials reporting no effects [30, 33] or in smaller field trials reporting moderate, variable effects [21, 32]. Beyond the facts that we (i) measured adult-mosquito density *via* active aspiration, not trapping, and (ii) used very simple dissemination stations with low-concentration (0.5% a.i.) PPF, these differences may reflect small trial size in [21, 32] or non-independence of intervention and control blocks in [30, 33]. In contrast, our current estimates of MD-PPF effects on adult-mosquito density are quantitatively below what would be expected if, as suggested by neighborhood- or town-scale trials using the same dissemination stations and PPF formulation, MD-PPF had reached most (> 90%) aquatic larval habitats and killed most (~ 70–90%) immature mosquitoes before adult emergence [25, 28]. It should be noted, first, that our IC was spatially isolated from the CC, but not from neighboring, untreated residential areas (Fig. 1); we therefore expected that mosquito immigration into the IC would weaken intervention effects. This was also the case in the study by Abad-Franch et al. [25] and probably in all other previous field trials of MD-PPF [19, 21, 23, 24, 26, 27, 30–33], with one exception, i.e. the only citywide study reported so far, in which mosquito immigration was particularly unlikely and MD-PPF had a particularly strong impact on local mosquito populations [28]. Secondly, we note that the intensity of the intervention was comparatively low in our trial, in the sense that we deployed ‘just’ 150 PPF dissemination stations (*vs* 1000 in [28]) at relatively low spatial densities (*vs* 100 spatially-clustered stations in [25]) and, importantly, serviced them every four weeks (*vs* fortnightly in [25, 28]).

We therefore stress that the effects of MD-PPF we report came about *in spite of* the spatial non-isolation of the IC *and* the relatively low intensity of the intervention including monthly servicing of PPF dissemination stations. This suggests that effective MD-PPF-based interventions may be even more readily scalable than indicated by previous trials using the same, very simple dissemination stations and PPF formulation we used here [25, 28]. Finally, and along these lines, it is clear that study-site peculiarities (in terms of, e.g. mosquito species composition or population density, availability of larval habitats, or climate), as well as other factors (e.g. dissemination-station design), may also contribute to variation among MD-PPF trial results. Our results come with the additional caveats that (i) our sampling dwellings were a roughly systematic, relatively small, nonrandom sample of the dwellings in the study clusters, and (ii) field and laboratory teams were not blinded to the intervention. The picture that is emerging, however, is one of overall support for the view of MD-PPF as a very promising addition to the urban-mosquito control toolkit [15–34, 56]. Because of the trial’s cluster-randomized controlled design and the thorough strategy used for data analysis, and in spite of its limitations, our study adds some of the strongest evidence yet in favor of this view.

**Fig. 5:**
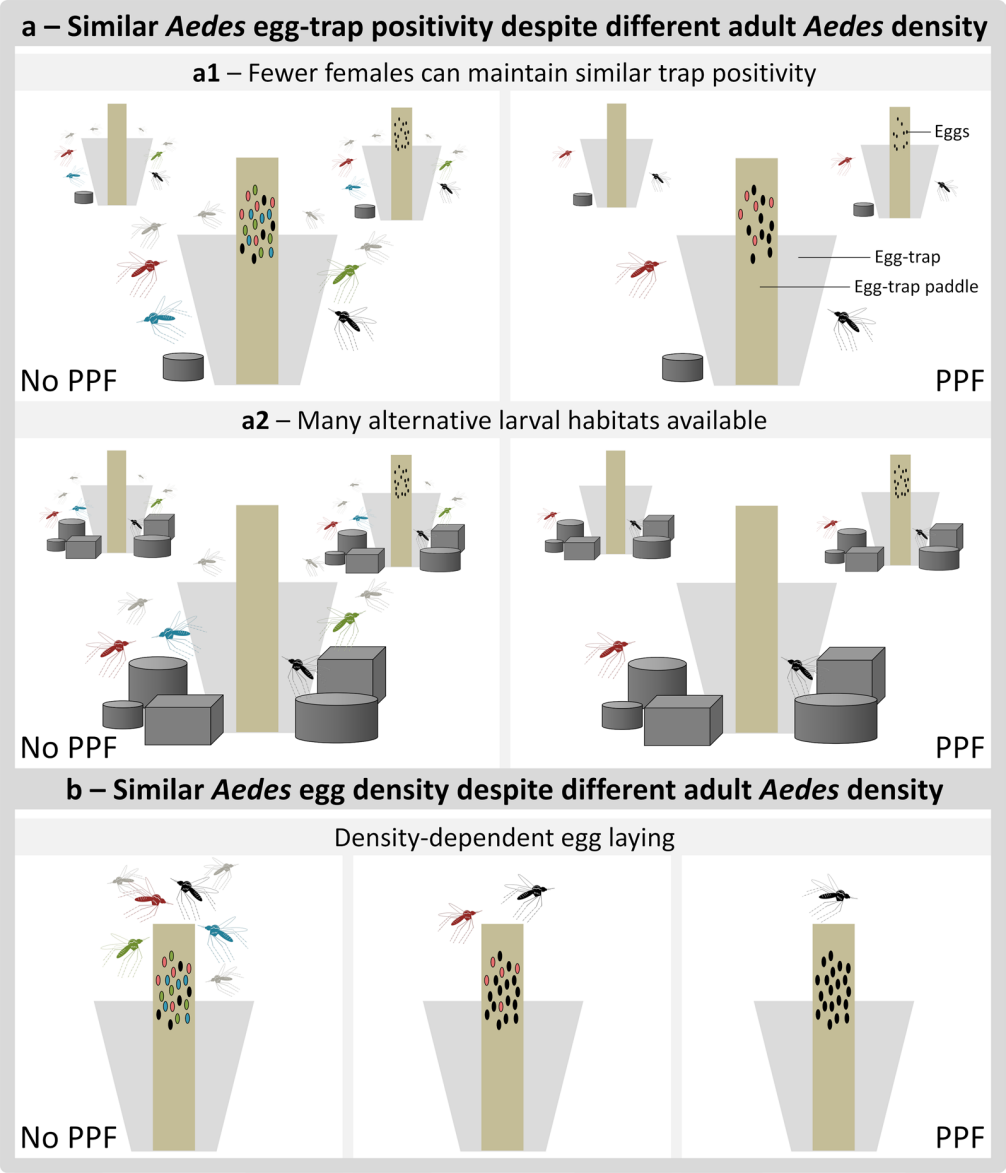
Three possible mechanisms underlying the observed mismatch between adult *Aedes aegypti* catches and egg-trap positivity (**a**) and egg density (**b**). **a1**: a small number of females may lay eggs in about as many sites as a large number of females; hence, egg-trap positivity remains largely unchanged; **a2**: if many alternative egg-laying sites are locally available, egg-traps may be relatively less attractive to *Aedes* females; hence, egg-trap positivity may be low regardless of adult-*Aedes* density; **b**: at high *Aedes* densities, females gather at ‘crowded’ egg-trap paddles and each female (black, blue, green, red) lays just a few eggs (left panel); at low adult densities, the earliest-arriving female (black) lays many eggs, and later-arriving females (red) lay just a few eggs (central panel); at very low *Aedes* densities, ‘lone’ females may lay many eggs each in otherwise vacant egg-trap paddles (right panel). *Abbreviation*: PPF, pyriproxyfen

One additional outcome of our CRCT was that *Aedes* egg-trap-based metrics failed to reflect the impact of MD-PPF on adult *Ae. aegypti* density as directly measured by mosquito aspiration (Tables 3 and 5, Figs. 3 and 4). Further, the observed baseline density of *Aedes* adult mosquitoes was clearly higher in the IC than in either the CC (both periods) or the IC during MD-PPF deployment, yet *Aedes* egg-trap positivity was not particularly high in the baseline IC data, and the number of *Aedes* eggs per egg-trap was actually the lowest we observed (see Tables 1 and 4). Three not mutually-exclusive hypotheses come to mind as plausible candidates to explain these observations. First, *Aedes* egg-trap positivity may remain largely unchanged if a small number of females can lay eggs in about as many sites as a substantially larger number of females [57] (Fig. 5). Secondly, low *Aedes* egg-trap positivity may be expected, irrespective of *Aedes* density, if so many suitable egg-laying sites are locally available that egg-traps are seldom chosen [37] (Fig. 5). Our field observations indicate that potential *Aedes* larval habitats, and in particular discarded trash items, may indeed have been more common in the IC than in the CC, but we did not measure this in a systematic way amenable to quantitative analysis. Finally, density-dependent egg-laying by *Aedes* females might yield a pattern of similar egg densities despite different adult abundances; this may occur, for example, if ‘lone’ *Aedes* females tend to lay many eggs in vacant egg-trap paddles, whereas females co-visiting a ‘crowded’ paddle tend to lay just a few eggs each [57–61] (Fig. 5). Whatever the actual underlying mechanism(s), our results suggest that the widely used egg-trap-based monitoring may measure poorly the impact of *Aedes* control; even if more costly, direct monitoring of the adult mosquito population is likely to provide a much more realistic and informative picture of intervention effects [5, 37–40, 62]. The fact that we found very little among-collector variation in mosquito-aspiration catches lends further support to this idea.

A final finding of our study was that ultra-low volume malathion spraying (ULV) had overall negligible effects on local mosquito populations, as shown by the fact that models including the ‘ulv’ covariate did not perform any better than models without it [48]. At most, we found some evidence that ULV may be associated with a very short-lived (about one-week) decrease of adult-mosquito densities [7–9, 14], possibly with a larger effect on *Ae. aegypti* than on *Cx. quinquefasciatus*. The seemingly ‘positive’ effect of ULV applied 8–30 days before sampling on *Ae. aegypti* (Additional file 1: Table S5) probably reflects the fact ULV is usually deployed when disease surveillance detects signals of local transmission of *Aedes*-borne viruses, an event that is presumably associated with high *Aedes* densities [62].

## Conclusions

In summary, our cluster-randomized controlled trial provided strong evidence that mosquito-disseminated pyriproxyfen (MD-PPF) can significantly reduce adult-mosquito densities at the urban-neighborhood scale. MD-PPF had a clear negative impact on both *Ae. aegypti* and *Cx. quinquefasciatus*; importantly, it did so in spite of (i) very likely immigration of adult mosquitoes from adjacent, non-treated areas and (ii) a rather long (four-week) time-lag between PPF dissemination-station servicing visits. On the other hand, we found that egg-trap-based indices may perform poorly at measuring *Aedes* control; direct monitoring of adult-mosquito populations (e.g. with aspirators or traps) is probably much more informative about intervention effects. If egg-traps are to remain widely used in routine surveillance and in the study of *Aedes* ecology and control, the mechanisms that underlie the mismatch between egg-trap data and adult-mosquito density should be further elucidated. Finally, and in line with previous reports, we found that ‘pulses’ of ULV space spraying had a very limited, short-lived effect on adult-mosquito densities. This report, in sum, adds important new findings to the growing body of evidence suggesting that MD-PPF can develop into a major tool for urban-mosquito control. The next, decisive step should be to dependably measure the impact of MD-PPF, alone or in combination with other tactics [5, 6], on mosquito-borne disease transmission in the community. Crucially, our results and those from previous trials strongly suggest that, if it is to protect people from infection and disease, MD-PPF will have to be deployed over whole neighborhoods or entire towns. Extensive efforts to test this hypothesis are currently underway in Brazil.

## Supplementary information

**Additional file 1: Table S1.** Weather covariate values. **Table S2.** Monthly adult-mosquito aspiration results. **Table S3.** Alternative models for the all-mosquito aspiration dataset. **Table S4.** ‘Full’ all-mosquito aspiration model with ULV. **Table S5.** Alternative models for the *Aedes aegypti* aspiration dataset. **Table S6.** ‘Full’ *Aedes* aspiration model with ULV. **Table S7.** Alternative models for the *Culex quinquefasciatus* aspiration dataset. **Table S8.** ‘Full’ *Culex* aspiration model with ULV. **Table S9.** Monthly *Aedes* egg-trap results. **Table S10.** Alternative models for the *Aedes* egg-trap dataset.

**Additional file 2: Dataset S1.** Adult-mosquito aspiration data are provided in the sheet named ‘Aspiration’, and *Aedes* egg-trap data in the sheet named ‘Aedes egg traps’. The file includes a ‘Readme’ sheet with the meaning of each variable.

**Additional file 3: Table S11.** Top-ranking (smallest-BIC) model for female adult-mosquito aspiration data analyzed separately. **Table S12.** Top-ranking (smallest-BIC) model for male adult-mosquito aspiration data analyzed separately.

## Data Availability

Data supporting the conclusions of this article are included in the article and its additional files.

## Abbreviations

PPF: pyriproxyfen
MD-PPF: mosquito-disseminated pyriproxyfen
CRCT: cluster-randomized controlled trial
CC: control cluster
IC: intervention cluster
BP: baseline period
IP: intervention period
ID: identity of each sampling dwelling
ULV: ultra-low volume insecticide spraying
a.i.: active ingredient
ha: hectare
GLMM: generalized linear mixed model
BIC: Bayesian information criterion
SE: standard error
SD: standard deviation
95% CI: 95% confidence interval
