# Supplementary material for: Measuring mosquito control: adult-mosquito catches vs egg-trap data as endpoints of a cluster-randomized controlled trial of mosquito-disseminated pyriproxyfen
Source: Parasit Vectors. 2020 Jul 14;13:352. doi: 10.1186/s13071-020-04221-z (PMC7362459; doi:10.1186/s13071-020-04221-z)
Supplement: Supplementary file 3 — Additional file 3: Table S11. Top-ranking (smallest-BIC) model for female adult-mosquito aspiration data analyzed separately. Table S12. Top-ranking (smallest-BIC) model for male adult-mosquito aspiration data analyzed separately. [file 13071_2020_4221_MOESM3_ESM.pdf]

Garcia et al. Measuring mosquito control: adult-mosquito catches *vs*. egg-trap data as endpoints of a cluster-randomized controlled trial of mosquito-disseminated pyriproxyfen. Additional file 3: Tables S11 and S12.

Table S11. Adjusted effects of mosquito-disseminated pyriproxyfen on female-mosquito catches (*Aedes aegypti* + *Culex quinquefasciatus*): top-ranking (smallest-BIC) generalized linear mixed model

Terms	Estimate	SE	CI_{95}	
			Lower	Upper
Fixed effects				
Intercept (CC, BP)[a]	0.138	0.401	−0.649	0.924
Intervention period (IP)[b]	−0.078	0.402	−0.867	0.711
Intervention cluster (IC)	−0.732	0.326	−1.371	−0.093
IP × IC[c]	−0.715	0.242	−1.190	−0.240
Temperature[d]	0.837	0.174	0.495	1.179
Random effects SD				
Dwelling ID	0.956	-	0.769	1.190
Month	0.553	-	0.372	0.822

[a] The intercept estimates the (log-scale) expected mean number of female mosquitoes caught per 10 min aspiration in the CC, in the typical dwelling and at typical temperatures, during the BP; the other fixed-effect slope coefficients estimate changes in this expectation associated with period, cluster, intervention, and temperature effects.
[b] Note that the model estimates a near-zero change in (log) mean female-catch as the CC entered the IP (but received no intervention); the estimated incidence rate ratio is $e^{-0.078}$ = 0.925, or a 7.5% decrease in mean female-catch, with the CI_{95} spanning zero.
[c] The 'IP × IC' interaction coefficient estimates the (log) change in expected mean female mosquito-catch that can be attributed to the intervention – deployment of 150 pyriproxyfen dissemination stations over 13 months (the intervention period 'IP') in the intervention cluster 'IC'. Here, the model estimates an $e^{-0.715}$ = 0.489 incidence rate ratio, indicating that the intervention resulted in a 100 − 48.9 = 51.1% reduction (CI_{95} 21.3–69.6%) of the expected mean female mosquito-catch.
[d] Specified as the (standardized) mean of minimum daily temperatures in the month before each sampling occasion ('tmin_m'); the original variable had mean = 17.39°C and SD = 1.73°C. Given our focus on estimating adjusted intervention effects, we considered weather covariates as confounders; 'tmin_m' yielded better-performing models, as measured by BIC scores, than other measures of temperature and rainfall.
Abbreviations: BIC, Bayesian information criterion; SE, standard error; CI_{95}, 95% confidence interval (lower/upper limits); CC, control cluster; BP, baseline period; IP, intervention period; IC, intervention cluster; SD, standard deviation; ID, identity of each sampling dwelling.

Table S12. Adjusted effects of mosquito-disseminated pyriproxyfen on male-mosquito catches (*Aedes aegypti* + *Culex quinquefasciatus*): top-ranking (smallest-BIC) generalized linear mixed model

Terms	Estimate	SE	CI_{95}	
			Lower	Upper
Fixed effects				
Intercept (CC, BP)[a]	−0.142	0.337	−0.801	0.518
Intervention period (IP)[b]	0.201	0.307	−0.400	0.801
Intervention cluster (IC)	−0.147	0.378	−0.887	0.593
IP × IC[c]	−1.451	0.297	−2.033	−0.868
Temperature[d]	0.655	0.125	0.409	0.900
Random effects SD				
Dwelling ID	1.057	-	0.831	1.343
Month	0.346	-	0.203	0.591

[a] The intercept estimates the (log-scale) expected mean number of male mosquitoes caught per 10 min aspiration in the CC, in the typical dwelling and at typical temperatures, during the BP; the other fixed-effect slope coefficients estimate changes in this expectation associated with period, cluster, intervention, and temperature effects.
[b] Note that the model estimates a small increase in (log) mean male-catch as the CC entered the IP (but received no intervention); the estimated incidence rate ratio is $e^{0.201}$ = 1.223, or a 22.3% increase in mean male-catch, with the CI_{95} spanning zero.
[c] The 'IP × IC' interaction coefficient estimates the (log) change in expected mean male mosquito-catch that can be attributed to the intervention – deployment of 150 pyriproxyfen dissemination stations over 13 months (the intervention period 'IP') in the intervention cluster 'IC'. Here, the model estimates an $e^{-1.451}$ = 0.234 incidence rate ratio, indicating that the intervention resulted in a 100 − 23.6 = 76.6% reduction (CI_{95} 58.0–86.9%) of the expected mean male mosquito-catch.
[d] Specified as the (standardized) mean of minimum daily temperatures in the month before each sampling occasion ('tmin_m'); the original variable had mean = 17.39°C and SD = 1.73°C. Given our focus on estimating adjusted intervention effects, we considered weather covariates as confounders; 'tmin_m' yielded better-performing models, as measured by BIC scores, than other measures of temperature and rainfall.
Abbreviations: BIC, Bayesian information criterion; SE, standard error; CI_{95}, 95% confidence interval (lower/upper limits); CC, control cluster; BP, baseline period; IP, intervention period; IC, intervention cluster; SD, standard deviation; ID, identity of each sampling dwelling.
